# Conformation Regulation of the X Chromosome Inactivation Center: A Model

**DOI:** 10.1371/journal.pcbi.1002229

**Published:** 2011-10-27

**Authors:** Antonio Scialdone, Ilaria Cataudella, Mariano Barbieri, Antonella Prisco, Mario Nicodemi

**Affiliations:** 1 Department of Computational and Systems Biology, John Innes Centre, Norwich, United Kingdom; 2 Center for Models of Life, Niels Bohr Institute, Copenhagen, Denmark; 3 Dipartimento di Scienze Fisiche, Università di Napoli “Federico II,” INFN, Napoli, Italy; 4 CNR Istituto di Genetica e Biofisica “B. Traverso”, Napoli, Italy; 5 Dipartimento di Scienze Fisiche, Università di Napoli “Federico II,” INFN, CNR-SPIN, Napoli, Italy; Ottawa University, Canada

## Abstract

X-Chromosome Inactivation (XCI) is the process whereby one, randomly chosen X becomes transcriptionally silenced in female cells. XCI is governed by the *Xic*, a locus on the X encompassing an array of genes which interact with each other and with key molecular factors. The mechanism, though, establishing the fate of the X's, and the corresponding alternative modifications of the *Xic* architecture, is still mysterious. In this study, by use of computer simulations, we explore the scenario where chromatin conformations emerge from its interaction with diffusing molecular factors. Our aim is to understand the physical mechanisms whereby stable, non-random conformations are established on the *Xic*'s, how complex architectural changes are reliably regulated, and how they lead to opposite structures on the two alleles. In particular, comparison against current experimental data indicates that a few key cis-regulatory regions orchestrate the organization of the *Xic*, and that two major molecular regulators are involved.

## Author Summary

In mammal female cells X-Chromosome Inactivation (XCI) is the vital process whereby one X, randomly chosen, is silenced to compensate dosage of X products with respect to males. XCI is governed by a region on the X, the X Inactivation Centre (*Xic*), which undergoes a sequence of conformational modifications during the process. The two *Xic* are exposed, though, to the same environment, and it is obscure how they attain different architectures. By use of computer simulations of a molecular model, here we individuate general physical mechanisms whereby random Brownian molecules can assemble chromatin stable architectures, reliably regulate conformational changes, and establish opposite transformations on identical alleles. In the case-study of the murine *Xic*, our analysis highlights the existence of a few key regulatory regions and molecular factors. It also predicts, e.g., the effects of genetic modifications in the locus, which are compared with current deletion/insertion experiments. The physical mechanisms we describe are rooted in thermodynamics and could be relevant well beyond XCI.

## Introduction

**Figure 1:**
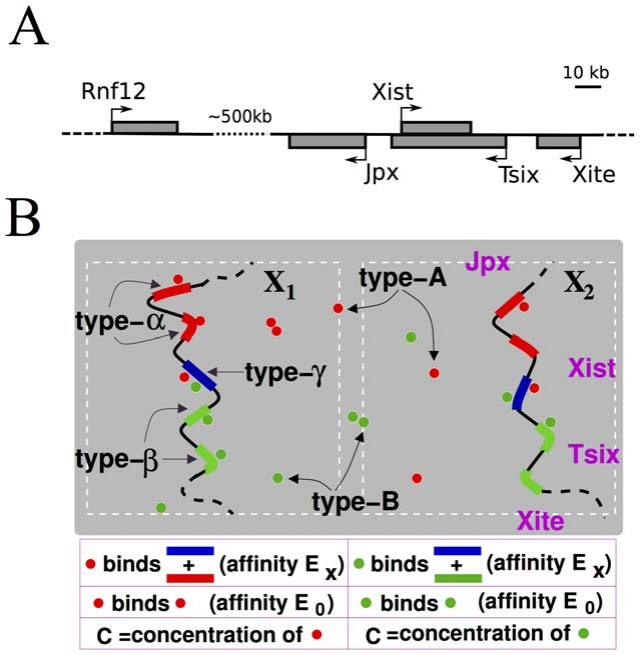
The model. **Panel A** is an illustration of the region of the X Inactivation Centre (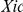) around the 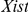 gene. The scheme in **panel B** zooms on the key regions of the two polymer model investigated here. Each polymer has two type-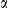 (red), two type-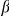 (green) and a type-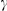 (blue) regions. Type-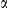 and type-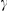 can be bridged by type-A molecules (red circles) with an affinity 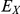; type-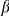 and type-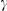 by type-B molecules (green circles). Each molecular species has a concentration 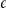. Type-A molecules have also a homotypic mutual interaction of affinity 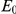, and similarly type-B ones. The presumptive mapping areas on the 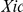 are also illustrated (right panel).

X-Chromosome Inactivation (XCI) is the vital process occurring in female mammalian cells whereby one randomly selected X is transcriptionally silenced to balance dosage with respect to males [1]–[4]. XCI is regulated by a region on the X chromosome, the X inactivation center (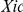), which encompasses a key group of neighboring non-coding genes (see Fig. 1.A) including, e.g., 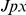, 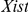, 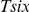 and 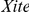 [1]–[4]. The fate of the X is determined by its 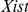 gene which is strongly upregulated on the future inactive X and repressed on the other X. In turn, 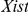 is negatively regulated by 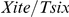, and positively regulated by 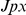, 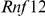, and other factors [5]–[7].

Before random XCI starts, a complex epigenetic program, coupling transcription and chromatin remodelling [8], [9] to pluripotency factors [10]–[12], produces a state where the 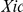 has the same spatial conformation on the two X chromosomes [13] and both 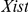 alleles are just weakly active. Upon XCI, an unknown symmetry breaking mechanism determines the opposite behaviour of the two 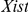, and induces alternative modifications of the three-dimensional conformation of their 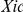 [13], [14]. Finally, on the designated inactive X further chromatin reorganizations occur as a heterochromatic compartment forms into which genes are recruited to be silenced [3], [15]. Several molecular factors are known to be involved in the process [3], [4], including noncoding transcripts, chromatin modifiers and organizers, such as CTCF (a Zn finger having arrays of binding sites on the *Xic*), Dnmt3a, Oct4 and other pluripotency factors [9]–[12], [16], [17]. Different models have been proposed to describe random XCI [18]–[22], but still none to elucidate its associated chromatin changes, whose nature remains mysterious.

To understand the principles of chromatin organization, within the murine 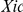 case study, here we explore the scenario where chromatin conformations emerge from its interaction with diffusing molecular factors. We discuss general physical mechanisms whereby random Brownian molecules can: *i)* succeed in establishing stable, non random conformations on the chromosomes; *ii)* reliably regulate specific conformational changes; and *iii)* produce opposite transformations on identical alleles exposed to the same environment (“symmetry breaking”). We investigate by computer simulations a schematic model consisting of two identical polymers which interact with a concentration of diffusing molecules (see Fig. 1.B). In the light of current 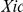 3C data [13], the model poses that along each polymer three types of regions exist type-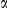, 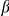 and 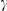) and predicts the existence of two types of regulatory molecules (type-A and B).

We show that the system thermodynamic stable states fall in distinct classes corresponding to different conformations. The polymers spontaneously select one of them according to molecule concentration/binding energy. Conformational changes are driven by thermodynamic phase transitions which act switch-like, regulated by given concentration/binding energy thresholds. The two polymers are exposed to the same environment, yet they can undergo alternative architectural modifications: we show that a symmetry breaking mechanisms is activated if the homotypic interaction between regulatory molecules rises above a threshold.

Comparison to experimental observations [1]–[5], [13], [21] suggests that the regions envisaged by the model can be approximately mapped along the 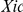 sequence as illustrated in Fig. 1.B, while type-A and B complexes could be related to an activating and a blocking regulator of 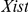.

## Model

We represent the relevant region of each X chromosome (see scheme in Fig. 1.B) by a standard model of polymer physics, a self-avoiding bead chain [23]. In the light of 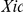 current 3C data [13], we pose that along each polymer there are, for simplicity, two type-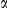 regions which have an array of binding sites for type-A Brownian molecular factors. Each polymer has also two type-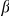 regions with binding sites for a different kind of molecular factors (type-B). Finally, the polymers have a type-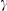 region whose binding sites can be bound by either type-A or B molecules. Thus, type-A molecules (resp. type-B) can bridge a type-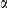 (resp. type-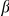) and a type-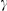 site. For simplicity, with no loss of generality, we consider the case where the two types of molecules have the same concentration, 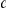, and the same affinity, 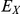, for all binding regions. Similarly, we assume that type-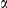 and type-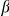 regions have the same number of binding sites, 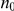, than type-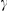. The value of 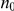 is fixed to have a total binding site number of the order of known 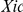 binding molecules. As CTCF is a general chromatin organizer which has been associated to XCI and its 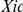 binding sites have been well characterized [17], we use it as an example (and set 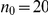). For simplicity, 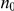 is here also the length of the intervening inert sequences between them. Type-A (resp. type-B) molecules can bind, with multiple valency, each other with affinity 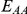 (resp. 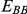); we set 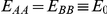 and, considering the number of binding domains of CTCF, the valency to four.

We investigate by Monte Carlo (MC) simulations the conformations of the system as they spontaneously emerge when the three control parameters, 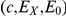, are varied. For computational purposes, the system lives in a cubic lattice with a lattice spacing 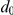, whose value corresponds to the typical size of a DNA binding site, and can be roughly estimated to be 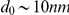. The volume concentration of molecules in our model, 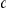, can be related to molar concentrations 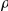: 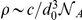, 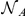 being the Avogadro number (details in Text S1). Thus, for instance, a typical nuclear protein concentration of 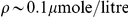 would correspond to 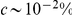. Below we consider concentrations in the range 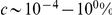 and binding energies in the weak biochemical scale (a few units in 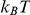). Finally, conversion of MC time unit to real time is obtained by imposing that the diffusion constant of our polymers is of the order of measured chromatin diffusion constants (see Text S1 for details).

## Results

### Establishing stable interactions

We first show that diffusing molecules can produce a looped conformation on each polymer where type-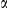 and type-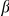 stably interact with type-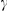 region. The process is based on a thermodynamic mechanism (a phase transition, in the thermodynamic limit) which acts switch-like when concentration/affinity of binding molecules rise above a threshold [24].

**Figure 2:**
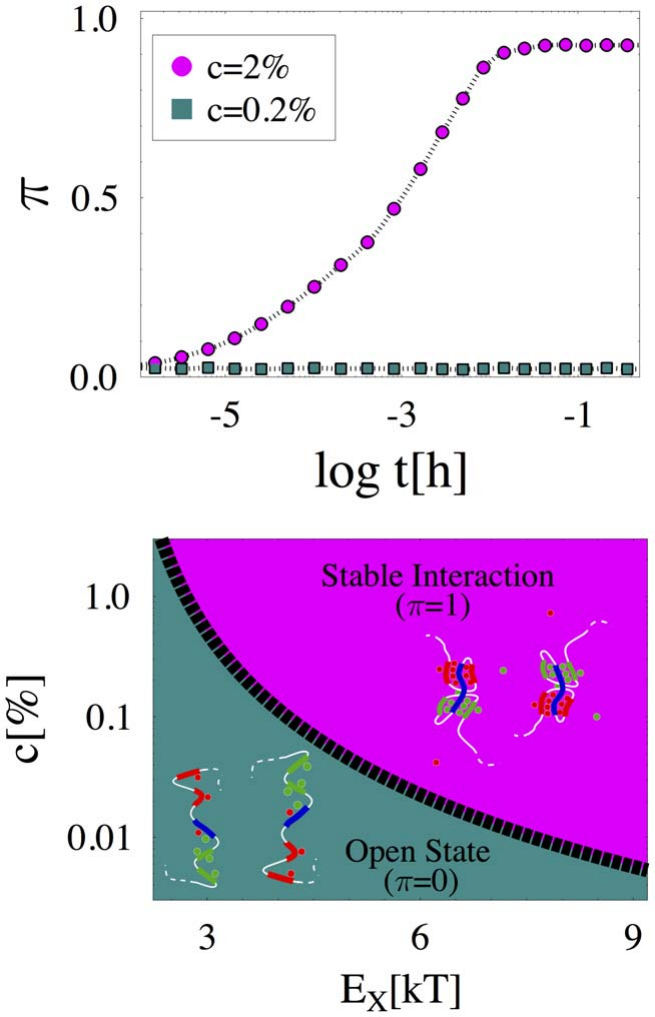
Conformational Switches and the establishment of stable interactions. **Top Panel** 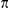 is the probability of type-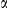 and type-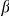 regions to loop stably onto type-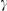 region. Its time evolution, from an initial open polymer conformation (see schematic representation in the Bottom Panel, “Open State”), is shown for two characteristic values of the concentration, 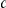 (here 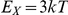 and 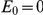). For 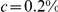, 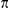 is zero at all times: neither type-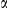 nor type-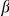 regions succeed in forming stable contacts with type-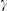, and the polymer conformation remains open. For 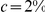, after a transient of the orders of minutes, 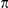 approaches one: a stable, looped conformation is established (see schematic picture in the Bottom Panel, “Stable Interaction”). **Bottom Panel** The conformation phase diagram in the 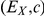 plane is shown (for 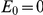): in the region below the sharp transition line, 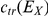 (black dashed line), the polymers are found in an open state; above 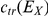, they exhibit a conformation change, symmetrical on the two polymers, as a stable interaction of type-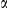 and type-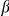 with type-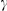 region is established.

Before describing our MC results in details, we illustrate the underlying mechanisms. A single, say, type A molecule forms a bridge between type-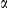 and type-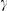 regions via the stochastic double encounter of the molecule with its binding sites. This is, though, an unlikely event, especially if molecule concentration, 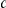 (or 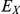, see below), is small. And the half-life of such a bridge is short when weak biochemical interactions are considered. Thus, on average the regions float away from each other (see pictorial representation in the bottom panel of Fig. 2, “Open State”). At higher 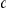 (or 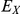), however, many a molecule can bind type-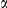/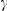 regions and stabilize the conformation via a positive feedback mechanism as their bridges reinforce each other and facilitate the formation of additional bridges. The concentration where such a positive feedback mechanism starts winning marks the threshold above which stable contacts are established (pictorial representation in the bottom panel of Fig. 2, “Stable Interaction”).

This pictorial scenario summarizes our MC results. For sake of simplicity, we consider first the case where molecule mutual interaction is turned off, 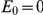, and set as initial configuration of the polymers a randomly open conformation. We measure the interaction order parameter, 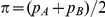, where 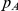 (resp. 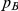) is the probability to have, on a polymer, a contact of a type-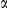 (resp. type-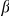) with type-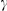 region. If neither type-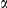 nor type-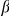 regions are in contact with 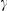, the order parameter is zero, 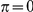; if only one pair is stably interacting then 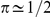; finally, 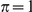 if both type-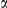 and type-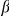 loops are established. Fig. 2 top panel shows the MC time evolution of 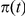 for two values of 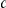: if 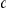 is small, 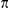 remains indefinitely close to zero, 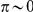, as no stable contact is statistically possible; instead, if 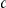 is high enough, 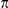 grows to a value close to one, 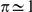, showing that both the type-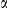 and 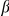 loops are formed.

### Conformation switch and sharp regulation

In the space of the control parameters, 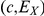, a sharp line separates the two regimes, as shown in Fig. 2 bottom panel: when 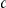 or 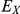 are small, contacts cannot be stable and 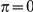; conversely, above the transition line the two loops conformation is reliably established on each polymer, and 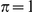. Such a line marks the boundary between two thermodynamic phases [25]: it corresponds to the point where the entropy loss due to loop formation is compensated by the energy gain obtained from the establishment of the corresponding bridges.

The discovery of such a switch-like behaviour can also explain how loop formation can be sharply and reliably regulated in the cell by increasing the concentration of specific molecular mediators or the affinity to their DNA target sites, e.g., by chromatin or molecule modifications.

The position of the transition line is also dependent on the number of available binding sites, 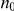, since, schematically, the overall binding energy scale is 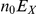. Thus, non-linear threshold effects in genetic deletion/insertions of the locus exist.

### Threshold values in real nuclei

From Monte Carlo results we can predict concentration (or energy) thresholds in real nuclei. For instance, *in vitro* measures of CTCF DNA binding energies give 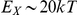, a typical value for TFs [26], [27]: an extrapolation from Fig. 2 then predicts a threshold 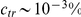, corresponding to a typical nuclear protein molar concentration 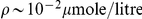 (see Text S1).

Finally, the mechanism leading to stable loop formation has to be fast enough to serve functional purposes. In our model we find that stable interactions are established on scales of the order of minutes (see Fig. 2 top panel and Text S1), a range consistent with biological expectations.

### Symmetry Breaking mechanism

**Figure 3:**
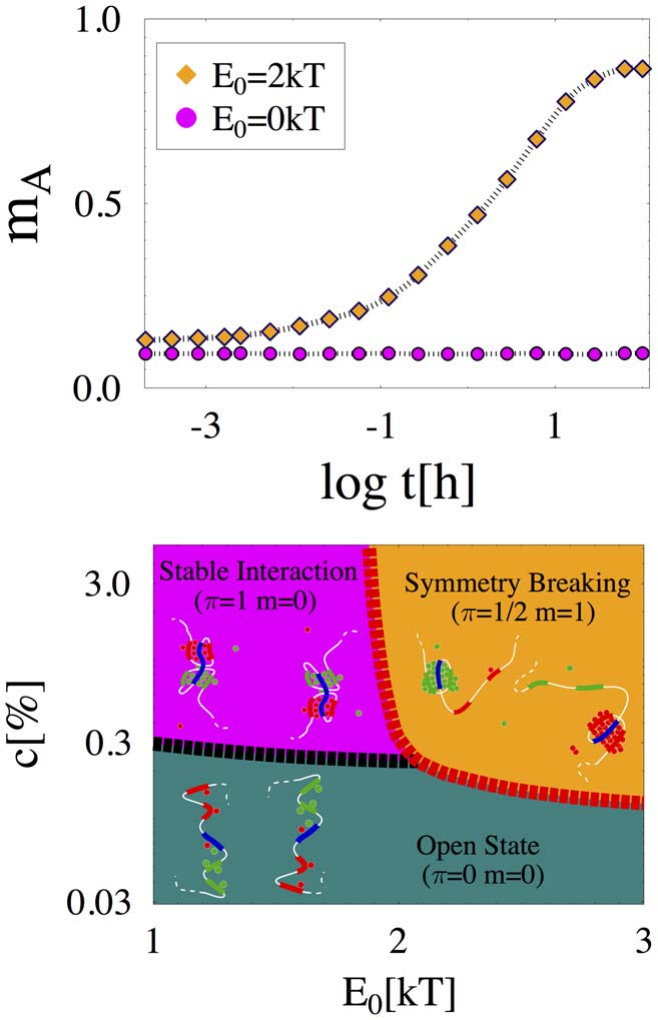
The Symmetry Breaking (SB) mechanism. **Top Panel** The SB parameter, 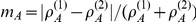, is the normalized average difference of type-A molecule density around type-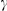 region of polymers 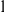 and 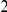. Its dynamics, from the initial symmetrical polymer looped state (as in the schematic picture in the Bottom Panel, “Stable Interaction”), is shown for two characteristic values of molecule homotypic interaction energy, 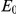 (here 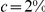 and 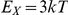). If 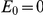, 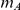 is close to zero: molecules are equally distributed around the polymers. If 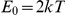, after a transient of about ten hours, 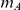 approaches one, i.e., either 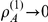 or 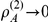: molecules have aggregated around only one of the polymers, and their binding symmetry is broken (as in the schematic picture in the Bottom Panel, “Symmetry Breaking”). **Bottom Panel** The phase diagram in the 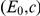 plane (for 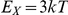) has three phases. If 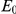 is below the transition line, 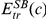 (red dashed line), the system is in one of its symmetric phases: the “Open State” phase (at low 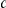) or the symmetrical “Stable Interaction” phase. If 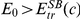, the conformational symmetry is broken (“Symmetry Breaking” phase): the type-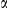 loop persists only on one randomly chosen polymer, and type-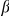 on the other.

The mechanism to induce conformational changes illustrated above acts “symmetrically” on the two polymers. Now we show that molecule homotypic interaction, 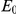, can break the polymer symmetry via a different thermodynamic mechanism. More precisely, if 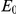 (and 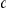, see below) is above a critical threshold, a single major aggregate of type A molecules and a single one of type B are formed because of homotypic binding cooperativity: in facts, the energy gain in forming a single cluster of A/B molecules (which maximizes the number of possible chemical bonds) compensates, if 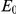 is large enough, the corresponding entropy reduction. The single, say, type A aggregate will then randomly bind just one polymer, leaving the other one “naked” (pictorial representation in the bottom panel of Fig. 3, “Symmetry Breaking”).

Type-A and B aggregates bind opposite polymers because A and B molecules compete for binding sites in the type-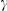 region. Hence, if a fluctuation increases the presence of, say, A molecules on one polymer, cooperativity tends to favor their assembling at that site and B molecules are expelled; in turn, the depletion of A around the other polymer favors the assembling of B molecules on it. On the polymer where the A cluster binds the type-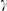 region, the B-related loci can no longer be stably linked, and their loop opens; the opposite situation happens on the other polymer.

The above scenario results from our MC simulations. We measured the symmetry breaking order parameter, 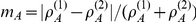, where 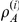 is the average local concentration of 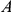 molecules around the type-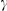 region of polymer 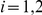. The 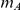 parameter is close to zero if an equal amount of A molecules is present around the two polymers, whereas it approaches one if the symmetry is spontaneously broken (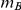 and 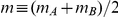 behave analogously). Fig. 3 top panel shows the time evolution of 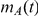 from an initial configuration corresponding to the symmetric state (schematic picture in the bottom panel of Fig. 3, “Stable Interaction”) where each polymer has two stable loops as seen before: if 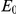 is small, 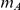 remains close to zero at all times and the system remains in a symmetric state; conversely, if 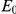 is high enough, 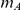 approaches one because A molecules reside mostly around just one, randomly chosen polymer and the symmetry is broken (schematic picture in the bottom panel of Fig. 3, “Symmetry Breaking”). The phase diagram of Fig. 3 bottom panel shows that the symmetry breaking mechanism is switch-like too: in the 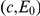 space, as soon as a narrow transition line is crossed the system switches from a symmetrical polymer state to a broken polymer symmetry state. More details are in the Text S1.

For sake of simplicity, we considered the case where the concentration/DNA affinity of molecules A and B are the same. However, such an assumption does not affect our general results. The only condition for the Symmetry Breaking and Configurational Switch mechanisms to be triggered is that concentration/interaction energy of both types of molecules rise above the appropriate threshold.

### Symmetry Breaking in real nuclei

As far as XCI is concerned, the predicted single B molecule aggregate is interpreted as an 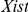 repressing factor (a Blocking Factor, BF) and designates the future active X. The A aggregate marks the X where 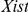 transcription is enhanced and is interpreted as an activating factor (AF). Importantly, the thresholds predicted by our theory for the symmetry breaking mechanisms also fall in the correct biochemical range (see above and Fig. 3 bottom panel).

The time scale required to break the symmetry in a real nucleus can depend on a number of details. Our MC provides, thus, only a very rough order of magnitude estimate. As shown in Fig. 3 top panel, such a time scale is predicted to be around 10 hours, a value of the order of the time required for XCI initiation.

In males other processes could intervene, yet it is easy to see how the same two factors mechanism can work, i.e., why the only X is usually bound by the B aggregate (and not by A) to repress 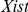. In fact, the affinities of A and B molecules for the type-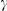 region are expected, in general, to be different: 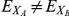. Hence, if 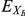 is larger than 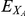, it is thermodynamically convenient that B molecules bind the X, a difference of a few units in 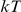 being sufficient to skew of orders of magnitudes the binding probability of A and B.

Finally, variants of the model can be considered to account for further biological details. For instance, additional molecular factors, or the effects on polymer colocalization can be discussed (see Text S1), but no relevant changes to the present scenario are found.

## Discussion

Our schematic model (Fig. 1.B) predicts that two kinds of molecular regulators, type-A and B molecules, interact with a set of specific regions along the polymers. Current 3C data [13] suggest that our type-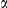 and type-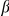 regions map respectively in the area 5′ and 3′ to 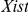, while the type-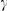 region is in between.

**Figure 4:**
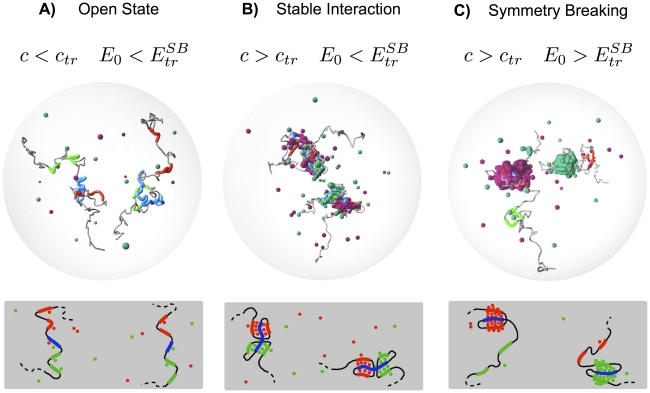
System states and transitions. The figure summarizes the system possible states and how they change by action of the Conformation and the Symmetry Breaking switch (top pictures are from MC simulations, bottom ones are schematic drawings). The switches have a thermodynamic nature and are regulated by increasing, e.g., 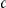 and 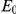 (i.e., molecule concentration and homotypic interaction) above precise threshold values, 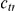 and 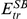. **A**) For 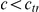 and 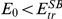, the polymers are found in a random open state. **B**) For 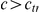, a conformation change is activated: type-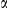 and type-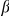 regions stably interact with type-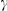, and a two loop conformation is established symmetrically on the two polymers. **C**) If 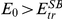, a symmetry breaking occurs as the type-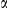 loop persists on one, randomly selected, polymer (where type-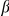 loop is released), whereas the other polymer takes the opposite conformation. This results from the self-assembling of a single major aggregate of type-A and of type-B molecules competing to bind to type-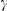 region.

We showed that in our model only three classes of stable conformational states exist (see Fig. 4 A,B,C). The system spontaneously falls in one of them, according to molecule concentration and homotypic interaction, 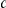 and 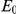. State changes are regulated by a “conformation” and by a “symmetry breaking” switch, related to two distinct thermodynamic phase transitions [25]. The switches are controlled by changing 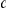 and 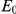 above/below specific threshold values. Their on/off nature can explain how a sharp regulation of nuclear architecture and stochastic choice of fate can be reliably obtained by simple strategies, such as protein upregulation or chromatin modification. Importantly, the model predicts energy/concentration thresholds which are in the expected biological range (weak biochemical energies, fractions of 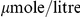 concentrations).

We now discuss how the present scenario can recapitulate in a unified framework important experimental results on XCI.

### Xic architecture, “counting” and “choice”

Before XCI, the 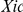 conformation is found to be identical on the two X's [13]: 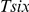 and 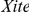 genes are looped onto a “buffer” region; similarly, 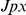, 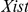 and the “buffer” form a second hub with 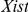. Upon XCI, on the future active X, the 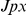-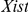-buffer hub opens while 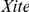 remains in contact with 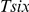. On the other X, instead, the 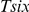-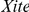 interactions is lost whereas 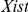 and 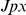 remain in contact.

Our model rationalizes how those elements are sharply regulated to recognize each other and to form stable interactions based on weak biochemical bonds. It can also explain how the same physical elements later at XCI spontaneously break the X symmetry. The molecular aggregate bound, in our model, to the type-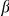 regions (which should encompass the 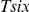-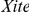 area) is interpreted as a factor related to 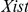 silencing (i.e., to its Blocking Factor, BF [1], [2], [4]) and designates the future active X; the different aggregate bound to type-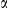 regions, encompassing the 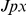 area of the other X would be linked to an 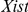 activating factor (AF) [5], [6], [22]. The link between architectural changes and choice of fate emerges here naturally.

During XCI establishment, the inactive X undergoes further architectural reorganization [3], [14], [15]. The mechanistic details of those conformational changes are still not understood, but they could involve mechanisms as those illustrated here.

Other interesting models have been proposed for “counting&choice” at XCI, but still none had focused on the 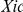 spatial organization, including our original Symmetry Breaking theory [19]. In the approach of ref. [21], each X chromosome is assumed to have an independent probability to initiate inactivation. Two competing factors exist: an X-linked XCI-activator and an XCI-inhibitor produced by autosomes. In a male XY cell the XCI-activator concentration is too low to initiate the inactivation of the only X; in female XX cells the initial XCI-activator concentration is, instead, above the threshold needed to start XCI. As soon as one X is inactivated, the XCI-activator concentration falls down to the levels found in males, and thus the other X remains active. A different model [22] poses that two types of sites are present on the X: “XCI-init” which is responsible for the initiation of inactivation of the X bearing it, and “XCI-repres” sites which inhibit the action of “XCI-init”. Each active X produces molecules, say 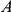 molecules, which bind to some autosomal sites. If these sites are saturated, the autosomes produce a set of molecules 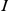, which, with a “Symmetry Breaking” mechanism [19], self-assemble into a single molecular factor and inhibit the activity of “XCI-repres” sites on one of the two X, determining its inactivation. As the availability of the 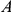 signal is reduced, it is no longer sufficient to saturate the autosomal receptors, and the remaining X remains active.

The mechanisms for conformational changes we discussed here are rooted in thermodynamics and are, thus, very robust to difference in molecular details. They could apply then to all the mentioned models for “counting&choice”. An interesting question concerns the applicability of those models to mammals other than mice. Important differences have emerged, for instance, between human and mice XCI [28], [29]. As stated above, the mechanisms we discussed for 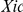 architecture in mice stem cells are very robust, yet data on other organisms are still too scarce to decide whether such mechanisms might apply elsewhere.

### Xic deletions/insertions and XCI

The phenotype of key deletions along the 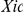 (see reviews in [1], [2], [4], [19], [22] and ref.s therein) can be explained by our model. The 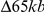 deletion [30] removes 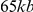 encompassing 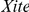 and part of 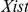/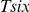. In heterozygous females the deleted X is always inactivated. In males it leads to the inactivation of the only X; the shorter the deletion considered within the 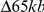 (see 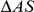, 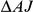, 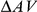, 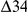 [31]), the smaller the fraction of ectopic X inactivations in a population.

Those deletions, in our model, map into sites where the 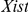 “blocking factor” (BF) binds (and blocks inactivation of that X): 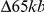 removes a large portion of binding sites, thus the deleted X has a strongly reduced affinity for the BF (w.r.t. the wild type X) which does not bind there; the shorter the deletion, the weaker the effect. So, in heterozygously deleted females a skewed random XCI occurs, whereas in males the only X can be inactivated. These deletions can also impact the formation of the BF itself because the involved regions possibly encode some of its components.

Heterozygous 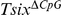 [32] and 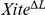 [33] deletions in females also result in the inactivation of the deleted X. Their homozygous counterpart produces, though, “aberrant counting/chaotic choice”, i.e., presence of two active or inactive X's in a fraction of the cell population [18]. While that cannot be easily rationalized by other models (see, e.g., [21]), in our framework it is originated simply because the BF can fail to bind at all [34].


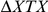
 is deletion including 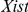, 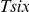 and 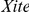, which in heterozygous causes a skewed XCI, as only the Wild Type X gets inactivated [21]. In the frame of our model 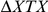 could have a double effect: on the one hand, it hinders the binding of the AF and BF to the deleted X, by removing a number of their binding sites; on the other it affects especially the BF, since it removes the 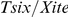 genes which are presumably linked to some of the BF components. Thus, the overall effect will be that while the deleted X remains active (as it lacks 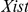), the BF is depleted and the AF wins the competition for binding the Wild Type chromosome, which is then inactivated.

Transgenic insertions are also interesting [35]. One of the predictions of our model is the highly non-linear effect of deletion/insertion, due to the “switch-like” nature of the underlying thermodynamic mechanism. The insertion experiments of ref. [35] support this view: long 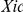 transgenes can cause inactivation on male ES cells only when they are present in multiple copies, while single insertions do not have appreciable effects. The outcome of other deletions/insertions, such as 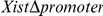 [36], 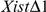-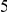 [37], 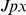 [5], 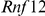 [6], etc., are similarly explained (see Text S1). XCI in diploid cells with more than two X and in polyploid cells [21] can be understood as well in our scenario (see Text S1), but additional biological hypotheses are required, since key pieces of information are still missing.

In summary, we illustrated physical switch-like mechanisms establishing conformational changes and symmetry breaking in a polymer model. For clarity, we included just the required minimal ingredients, but our model can accommodate more realistic molecular details. It can be mapped into the 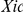 region of X chromosomes to explain their complex self-organization and other important aspects of random XCI, such as the deep connection between 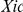 architectural changes and 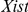 choice of fate, reconciling within a single framework a variety of experimental evidences. The on-off character of the underlying mechanisms can also explain how sharp and reliable regulation of XCI can be attained by simple strategies, such as gene upregulation or chromatin modification.

It supports a picture where random XCI could be governed by a few core molecular elements and basic physical processes. Two main groups of molecular factors are envisaged to control the process and to produce an activating and a blocking factor for 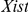. The specific polymer regions in our model emerge as key cis-regulators which orchestrates functional contacts along the 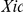. Experiments targeted at that area could test their role. The model also predicts threshold effects of, e.g., genetic deletions of the regulatory regions.

The precise nature of factors and sequences involved at XCI could differ from the minimal one considered here, yet the thermodynamic mechanisms we discussed are robust and independent of the specific molecular details. Similar mechanisms could be, thus, relevant to XCI and, more generally, to other nuclear processes requiring, for example, chromatin spatial reorganizations [38]–[40] or alternative choices [41].

## Supporting Information

Text S1Supplementary text and figures covering the following topics: additional details on the model, polymer colocalization, effects of 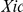 Deletion/Insertion experiments on XCI and XCI process in cells with more than two X's.(PDF)

## References

[1] Avner P, Heard E (2001). X-Chromosome Inactivation: counting, choice and initiation.. Nat Rev Genet.

[2] Wutz A, Gribnau J (2007). X inactivation Xplained.. Curr Opin Genet Dev.

[3] Chow J, Heard E (2009). X inactivation and the complexities of silencing a sex chromosome.. Curr Opin Cell Biol.

[4] Lee JT (2009). Lessons from X-chromosome inactivation: long ncRNA as guides and tethers to the epigenome.. Genes Dev.

[5] Tian D, Sun S, Lee JT (2010). The long noncoding RNA, Jpx, is a molecular switch for X chromosome inactivation.. Cell.

[6] Jonkers I, Barakat TS, Achame EM, Monkhorst K, Kenter A (2009). RNF12 is an X-Encoded dose-dependent activator of X chromosome inactivation.. Cell.

[7] Chureau C, Chantalat S, Romito A, Galvani A, Duret L (2011). Ftx is a non-coding rna which affects xist expression and chromatin structure within the x-inactivation center region.. Hum Mol Genet.

[8] Navarro P, Pichard S, Ciaudo C, Avner P, Rougeulle C (2005). Tsix transcription across the xist gene alters chromatin conformation without affecting xist transcription: implications for x-chromosome inactivation.. Genes Dev.

[9] Sado T, Hoki Y, Sasaki H (2005). Tsix silences xist through modification of chromatin structure.. Dev Cell.

[10] Navarro P, Chambers I, Karwacki-Neisius V, Chureau C, Morey C (2008). Molecular coupling of xist regulation and pluripotency.. Science.

[11] Donohoe ME, Silva SS, Pinter SF, Xu N, Lee JT (2009). The pluripotency factor Oct4 interacts with Ctcf and also controls X-chromosome pairing and counting.. Nature.

[12] Navarro P, Oldfield A, Legoupi J, Festuccia N, Dubois A (2010). Molecular coupling of tsix regulation and pluripotency.. Nature.

[13] Tsai CL, Rowntree RK, Cohen DE, Lee JT (2008). Higher order chromatin structure at the X-Inactivation center via looping DNA.. Dev Biol.

[14] Splinter E, de Wit E, Nora EP, Klous P, van de Werken HJG (2011). The inactive X chromosome adopts a unique three-dimensional conformation that is dependent on xist rna.. Genes Dev.

[15] Chaumeil J, Le Baccon P, Wutz A, Heard E (2006). A novel role for xist rna in the formation of a repressive nuclear compartment into which genes are recruited when silenced.. Genes Dev.

[16] Pugacheva EM, Tiwari VK, Abdullaev Z, Vostrov AA, Flanagan PT (2005). Familial cases of point mutations in the XIST promoter reveal a correlation between CTCF binding and pre-emptive choices of X chromosome inactivation.. Hum Mol Genet.

[17] Donohoe ME, Zhang LF, Xu N, Shi Y, Lee JT (2007). Identification of a Ctcf cofactor, Yy1, for the X chromosome binary switch.. Mol Cell.

[18] Lee JT (2005). Regulation of X-chromosome counting by Tsix and Xite sequences.. Science.

[19] Nicodemi M, Prisco A (2007). Symmetry-breaking model for X-chromosome inactivation.. Phys Rev Lett.

[20] Augui S, Filion GJ, Huart S, Nora E, Guggiari M (2007). Sensing X chromosome pairs before X Inactivation via a novel X-pairing region of the Xic.. Science.

[21] Monkhorst K, Jonkers I, Rentmeester E, Grosveld F, Gribnau J (2008). X Inactivation counting and choice is a stochastic process: evidence for involvement of an X-linked activator.. Cell.

[22] Starmer J, Magnuson T (2009). A new model for random X chromosome inactivation.. Development.

[23] Doi M, Edwards S (1986). The Theory of Polymer Dynamics.

[24] Nicodemi M, Prisco A (2009). Thermodynamic pathways to genome spatial organization in the cell nucleus.. Biophys J.

[25] Chandler D (1987). Introduction to Modern Statistical Mechanics.

[26] Quitschke WW, Taheny MJ, Fochtmann LJ, Vostrov AA (2000). Differential effect of zinc finger deletions on the binding of CTCF to the promoter of the amyloid precursor protein gene.. Nucleic Acids Res.

[27] Renda M, Baglivo I, Burgess-Beusse B, Esposito S, Fattorusso R (2007). Critical DNA binding interactions of the insulator protein CTCF: a small number of zinc fingers mediate strong binding, and a single finger-DNA interaction controls binding at imprinted loci.. J Biol Chem.

[28] Okamoto I, Patrat C, Thépot D, Peynot N, Fauque P (2011). Eutherian mammals use diverse strategies to initiate x-chromosome inactivation during development.. Nature.

[29] van den Berg IM, Galjaard RJ, Laven JSE, van Doorninck JH (2011). Xci in preimplantation mouse and human embryos: first there is remodelling….. Hum Genet.

[30] Clerc P, Avner P (1998). Role of the region 3′ to Xist exon 6 in the counting process of X-chromosome inactivation.. Nat Genet.

[31] Vigneau S, Augui S, Navarro P, Avner P, Clerc P (2006). An essential role for the DXPas34 tandem repeat and Tsix transcription in the counting process of X chromosome inactivation.. Proc Natl Acad Sci U S A.

[32] Lee JT, Lu N (1999). Targeted mutagenesis of Tsix leads to nonrandom X inactivation.. Cell.

[33] Ogawa Y, Lee JT (2003). Xite, X-Inactivation intergenic transcription elements that regulate the probability of choice.. Mol Cell.

[34] Nicodemi M, Prisco A (2007). Self-assembly and DNA binding of the blocking factor in X chromosome inactivation.. PLoS Comput Biol.

[35] Heard E, Mongelard F, Arnaud D, Avner P (1999). Xist yeast artificial chromosome transgenes function as X-inactivation centers only in multicopy arrays and not as single copies.. Mol Cell Biol.

[36] Penny GD, Kay GF, Sheardown SA, Rastan S, Brockdorff N (1996). Requirement for xist in x chromosome inactivation.. Nature.

[37] Marahrens Y, Panning B, Dausman J, Strauss W, Jaenisch R (1997). Xist-deficient mice are defective in dosage compensation but not spermatogenesis.. Genes Dev.

[38] Lanctôt C, Cheutin T, Cremer M, Cavalli G, Cremer T (2007). Dynamic genome architecture in the nuclear space: regulation of gene expression in three dimensions.. Nat Rev Genet.

[39] Misteli T (2007). Beyond the sequence: cellular organization of genome function.. Cell.

[40] Fraser P, Bickmore W (2007). Nuclear organization of the genome and the potential for gene regulation.. Nature.

[41] Gimelbrant A, Hutchinson JN, Thompson BR, Chess A (2007). Widespread monoallelic expression on human autosomes.. Science.

