# Supplementary material for: Conformation Regulation of the X Chromosome Inactivation Center: A Model
Source: PLoS Comput Biol. 2011 Oct 27;7(10):e1002229. doi: 10.1371/journal.pcbi.1002229 (PMC3203058; doi:10.1371/journal.pcbi.1002229)
Supplement: Text S1 — Supplementary text and figures covering the following topics: additional details on the model, polymer colocalization, effects of Deletion/Insertion experiments on XCI and XCI process in cells with more than two X's. (PDF) [file pcbi.1002229.s001.pdf]

Conformation Regulation of the X Chromosome Inactivation Center: a Model

Text S1

Antonio Scialdone[1], Ilaria Cataudella[2], Mariano Barbieri[3], Antonella Prisco[4], Mario Nicodemi[5,*]

1 Dept. of Computational and Systems Biology, John Innes Centre, Norwich, UK

2 Center for Models of Life, Niels Bohr Institute, Copenhagen, Denmark

3 Dip. di Scienze Fisiche, Univ. di Napoli "Federico II", INFN, Napoli, Italy

4 CNR Inst. Genet. and Biophys. "B. Traverso", Napoli, Italy

5 Dip. di Scienze Fisiche, Univ. di Napoli "Federico II", INFN, CNR-SPIN, Napoli, Italy

∗ E-mail: mario.nicodemi@na.infn.it

Contents

I. Main model and its simulations 3

A. Model Parameters and Computer Simulations 3

B. Further details on the Symmetry Breaking processes 5

C. Variants and Applications 5

II. Polymer Colocalization 7

A. Description of the Model 7

B. Dynamics of colocalization 8

C. The phase diagram 10

D. Colocalization of X chromosomes at XCI 11

III. Effects of Xic Deletion/Insertion on XCI 13

IV. Cells with more than two X's 18

References 25

I. MAIN MODEL AND ITS SIMULATIONS

We discuss here in more details the physical model of the Xic considered in the Main Text. We describe the relevant Xic sequence on each X chromosome by a standard model of polymer physics, a self-avoiding bead chain [1]. Along each polymer there are two type-α regions which have binding sites for type-A Brownian molecular factors. Type-A molecules can bind once those polymer beads with a chemical affinity, E_A, and are present in a concentration, c_A. Analogously, each polymer includes two type-β regions which have binding sites for a different kind of molecular factors (type-B) with affinity E_B and concentration c_B. Finally, the polymers have a type-γ region with binding sites for both type-A and B molecules. For simplicity, we consider the symmetric case where $c_A = c_B \equiv c$ and $E_A = E_B \equiv E_X$, with no loss of generality of our results. Similarly, we assume that the number of binding sites, n_0, for the molecular factors is the same for all region types. The value of n_0 is chosen to have a total number of binding sites of the order of those found experimentally in the locus for CTCF (here $n_0 = 20$) [2]. Type-A (resp. type-B) molecules can bind, with multiple valency, each other with an affinity E_{AA} (resp. E_{BB}). For simplicity, we set $E_{AA} = E_{BB} \equiv E_0$ and, considering the number of binding domains available on a CTCF molecule, the valency to four.

Summarizing, our physical model includes two identical self-avoiding polymers interacting with two kind of regulatory molecules (type-A and B), as also described in the Main Text. Along the polymers there are specific regions: type-α, β and γ. On the type-γ region either A or B molecules can bind. Type-A (resp. B) molecules can also bind type-α (resp. type-β) region. Each kind of molecule has a concentration c and an affinity, E_X, for its corresponding binding sites on the polymer. Molecules of type A can also bind each other, with a homotypic affinity E_0, and similarly the B molecules.

A. Model Parameters and Computer Simulations

The system is investigated by Metropolis Monte Carlo (MC) simulations [3]. For computational purposes, the system lives on a cubic lattice with lattice spacing, d_0. The value of d_0 corresponds to the typical size of a DNA binding site and can be roughly estimated to be about three orders of magnitude smaller than the nucleus diameter, say, $d_0 \sim 10nm$ (i.e., a

DNA sequence of about $\sim 30bp$). To reduce computation time, we do not simulate the whole nuclear space, but only consider a cubic lattice region of linear size L (in units of d_0) around each polymer; type-A and B molecules are allowed to diffuse from one to the other region. Below, we use lattices with $L = 32$ and periodic boundary conditions. Polymer chains have $n = 128$ beads, but we explored the range $L, n = 32 \div 256$ to check the robustness of our results. Averages run over up to 10^3 simulations from different initial configurations.

The fraction, c, of molecules per lattice site is related to their molar concentration, ρ: $\rho \sim c/d_0^3 \mathcal{N}_A$, where $\mathcal{N}_A$ is the Avogadro number. A typical nuclear protein concentration $\rho \sim 0.1\mu$mole/litre would correspond to $c \sim 0.01\%$: below we consider the range $c \sim 10^{-4} - 10^0\,\%$.

Diffusing particles (molecules and polymer beads) randomly move from one to a nearest neighbor vertex on the lattice, where no more than one particle can be present at a given time (single occupancy). Polymers obey a non-breaking constraint: two proximal beads can sit either on next or nearest next neighboring lattice sites. Chemical interactions are only permitted between nearest neighbor particles. In our MC simulations, the probability of a particle to move to a neighboring empty site is proportional to the Arrhenius factor $r_0 \exp(-\Delta E/kT)$, where ΔE is the energy change in the move, k the Boltzmann constant and T the temperature. The prefactor r_0 is the bare kinetic rate and gives the scale to convert Monte Carlo to real time: the Monte Carlo time unit, $\tau_0 = r_0^{-1}$, is the time to update once, on average, all the particles of the system [3].

We set r_0 here by imposing that the polymer diffusion coefficient, D, has the same order of magnitude than real mammalian DNA loci. We exploit the defining relation: $D = \langle \Delta s^2 \rangle / 6\tau_0$, where $\langle \Delta s^2 \rangle$ is the mean square displacement (expressed in units of d_0) of the polymer center-of-mass per unit MC time. In our system we find values around $\langle \Delta s^2 \rangle = 1.5 \cdot 10^{-3}\, d_0^2$. The order of magnitude of measured diffusion constants of, e.g., human DNA loci is $D \sim 1\mu m^2/hour$ [4]. Thus we obtain $\tau_0 \sim 90\mu s$, a value falling well within the range of known inverse biochemical kinetic constants [5].

Real dynamics is only very schematically captured by MC simulations. MC is considered, though, to well describe the general long time evolution of a system dominated by Brownian processes [3].

B. Further details on the Symmetry Breaking processes

Here we provide further details on the process leading to the polymer symmetry breaking. We focus on the 'Symmetry Breaking' phase of the phase diagram described in the Main Text. Fig. S1 shows the dynamics of a single run, from an initial configuration corresponding to a symmetric looped state (see Main Text), of the density of type-A and B molecules, $\rho_A^{(1)}(t)$ and $\rho_B^{(1)}(t)$, around the type-γ region of polymers 1: in the example pictured, the density of A strongly increases around polymer number 1, whereas $\rho_B^{(1)}$ goes approximately to zero. There is an initial transient when $\rho_A^{(1)}$ and $\rho_B^{(1)}$ behave similarly, yet $\rho_A^{(1)}$ later prevails, as soon as a fluctuation favoring the A's becomes spontaneously amplified. Thus, type-γ region on polymer 1 is depleted of B molecules and the loop they hold is released (i.e., the type-β region contacts opened). In Fig.S1 this is also illustrated by the interaction probabilities of type-α and type-β with the type-γ region on polymer 1, $p_A^{(1)}(t)$ and $p_B^{(1)}(t)$, which are reported for the same example. Interestingly, to produce the 'two loops' conformation and the later alternative architectural changes on the polymers, the model naturally requires the existence of at least two kinds of molecular factors (type-A and B).

C. Variants and Applications

In our model once a symmetry breaking molecular aggregate has bound its target on one polymer, the homologous open region on the other can diffuse and also bind the aggregate, leading to polymer colocalization. That occurs on a much longer time scale because it involves relocation of entire polymers (see next section). However, the occurrence of colocalization, or lack thereof, depends on the specific details of the system. For instance, we considered the case where type-A and B molecules can bind a polymer bead only if they are not previously bound to other molecules: such a variant leaves our scenario on conformational modifications and symmetry-breaking unaltered, but results in lack of polymer colocalization.

Experiments have shown that X Chromosome Inactivation (XCI) involves the colocalization of the two Xic's [6–8] around the time when $Xist$ expression is upregulated on one X and downregulated on the other. As in real nuclei a variety of intervening events and complications can arise, it is not straightforward to link X colocalization to the processes

described in our model; yet, thermodynamic mechanisms in the class of those described here [9, 10] could be the underlying driving force.

Finally, variants of the model can be considered to account for other complications. For instance, additional molecular factors can be involved, as those turning on A and B molecules homotypic interaction, E_0, as discussed in the next section, but no relevant changes to the present scenario are found.

II. POLYMER COLOCALIZATION

In this section we discuss the properties of a variant of the model considered in the main text to illustrate how the two polymers in the system can colocalize. We also discuss the role of additional molecular factors which might be involved, as those enhancing molecule-polymer interactions.

Since we account here for these additional elements, to make computation feasible we illustrate the mechanism for just one of the polymer regions of the model of the main text (say, only type-α). Our system here comprises two polymers and two different species of molecules: type-1 molecules can bind to the polymers, whereas type-2 molecules cannot directly bind the polymers but are capable to interact with each other and with type-1 molecules (see fig. S2).

We show that in this system a Symmetry Breaking Mechanism exists: when the concentration/mutual affinity of the molecules overcome a threshold, a stable molecular cluster of type-1/type-2 molecules is formed and binds to only one of the two identical polymers, randomly chosen. Then, we show that such a Symmetry Breaking mechanism can eventually produce the colocalization of the two polymers. However, the time scale for the colocalization process is much longer than the Symmetry Breaking time scale, as it requires that the entire polymers diffuse to encounter each other (see snapshots in Fig.S4).

A. Description of the Model

The model we consider is schematically pictured in Fig. S2. For sake of simplicity, we describe the two DNA polymers as two directed bead chains [1], i.e. their tips are bound to move on the top and bottom surfaces of the system volume. They comprise n beads which randomly move under a “non-breaking” constraint: two proximal beads can sit only in the next or nearest next neighboring lattice sites. The polymer beads can bind to a concentration c_1 of diffusing molecules (type-1), with energy E_p (see Fig. S2). These molecules can form a bridge between the beads of the different polymers and also bind (energy E_{12}), at most, two molecules of a different species (type-2), having a concentration c_2, which, in turn, have multiple valency mutual interactions (energy E_{22}).

The polymers as well as the molecules move in a lattice with a spacing constant, d_0, which, as in the main text, is of the order of the size of molecule DNA binding sites. Lattice models are well established in polymer physics [1] as they allow to circumvent the problem of computation feasibility, by permitting comparatively faster simulations with respect to off-lattice systems. We used a lattice with periodic boundary conditions having dimensions $L_x = 2L$, $L_y = L$ and $L_z = L$ in units of d_0, the lattice space constant. In the simulations here discussed $L = 16$, but we checked the results with L in the range $16 \div 64$. Each particle, i.e., a polymer bead as well as a molecule, occupies a single lattice site. Different particles are not allowed to sit at the same time on the same lattice site.

We run Monte Carlo (MC) computer simulations of this system, by using the Metropolis algorithm [3]. In this variant of the model, we fixed the time scale by imposing that the time scale to break the symmetry is the same we found in the model discussed in the main text. We take into account the faster dynamics of the molecules with respect to the DNA loci by fixing a molecule diffusion constant $5 \cdot 10^3$ times larger than that of the DNA loci, as experiments suggest (see [11] for typical diffusion constants of nuclear proteins and [4] for DNA loci). MC runs are up to 10^9 MC steps long and the averages are made over up to 1024 independent runs.

B. Dynamics of colocalization

We studied the dynamical and equilibrium properties of this system. We show that while at low concentration/affinity the molecules are equally distributed around the two polymers, at high concentration/affinity a unique molecular cluster is formed around one of the two polymers, and so the binding symmetry of the polymers is broken. Later on, the couple of polymers colocalizes at one order of magnitude longer time scale, with the molecular cluster that binds to both polymers and keeps them locked together. The colocalization as well as the symmetry breaking are on/off phenomena, as they occur only when the molecular affinity/concentration overcome certain threshold values, that fall well within the biologically relevant range of values.
To show this, we measured the pairing probability of the polymers p, namely the fraction of the polymer couples that are at a distance d less than $3d_0$. The average distance d between

the polymers is defined as: $d = \frac{1}{n}\sum_{i=1}^{n}\langle r(z_i)\rangle$, with $\langle r(z_i)\rangle$ being the distance between the polymer bead at height z_i, averaged over different simulations and over all the n polymer beads.

We monitored the symmetry between the polymers by measuring the order parameter $m = \left(|\rho_2^{(1)} - \rho_2^{(2)}|\right) / \left(\rho_2^{(1)} + \rho_2^{(2)}\right)$, where $\rho_2^{(i)}$, $i = \{1, 2\}$, is the density of type-2 molecules around the $i-$th polymer (the corresponding quantity for the type-1 molecule showed the same behaviour). A $m = 0$ indicates that, on average, the same number of type-2 molecules is found around the two polymers; conversely, a $m \sim 1$ signals that a single type-2 cluster is built around one of the two polymers, and so that the symmetry has been broken.

The behaviour of the system at above threshold values of the molecule concentration/affinity, is shown in fig. S3: it illustrates the pairing probability p (blue circles) and the order parameter m (orange squares) as function of the time t (here $E_p = E_{12} = E_{22} = 3kT$, $c_1 = 1\%$ and $c_2 = 4\%$). The initial configuration is with the molecules randomly distributed in the lattice and the polymers at a distance $d = L$.

The symmetry-breaking order parameter m starts from its random initial value close to 0, and, after a time $t \sim 1h$, increases at $\sim 80\%$, signaling that a single cluster of molecules is found only around one of the two polymers, and so that the binding symmetry has been broken. This happens while the polymers are still far from each other ($p \sim 0$), as it relies on the diffusion of the molecular binders. However, the polymers diffuse themselves, although with a comparatively lower diffusion constant; and as soon as they get closer, the cluster of molecules that originally was around only one polymer, now embraces both the polymers and keeps them colocalized: this is signalled by the increase of the pairing probability p to 100% and the corresponding decrease of m, that simultaneously occur after a time which is one order of magnitude larger than the time required for the symmetry breaking.

Fig. S4 helps visualizing the different stages of the system evolution: it shows a 2D projection of the lattice in a single run at three time frames. The green spots are the polymer centre-of-mass, and the density of molecules is represented in color scale (blue areas correspond to low densities, red to high densities). Initially the molecules are randomly distributed in the lattice (upper panel), but soon a unique cluster of molecules is seen around one of the two polymers (middle panel). The final colocalization of the polymers with the molecular cluster that includes both of them is clearly visible in the bottom panel.

C. The phase diagram

Different combinations for the values of the concentration/affinity of the molecules were tested. Interestingly, as discussed in the main text, we observed that the assembling of the molecular cluster around a polymer (and the following polymer colocalization) occurs in a "switch-like" fashion, namely only when the concentration/affinity of type-1 and type-2 molecules rise above a threshold, as a result of a mechanism that, in the thermodynamic limit, is a phase transition.

The physical reason for this behaviour is the following. The formation of a single aggregate of type-2 molecules is, in general, prevented by entropy: molecules tend to spread in the entire available volume rather than moving all in a given place. A single aggregate, however, allows to maximize the number of possible chemical bonds between type-2 molecules. Thus, if the bond energy is large enough, the total energy gain in forming a single aggregate compensates the related entropy reduction. In such a case, the system thermodynamic equilibrium state corresponds to the formation of a single aggregate [12].
More in details, type-2 molecules, with their reciprocal affinity, are responsible for the assembling of the single molecular cluster whereas type-1 molecules mediate the interaction between the polymers and the cluster. Thus, at low concentration/affinity of type-2 molecules, no cluster is going to form and the symmetry cannot be broken. Note that the colocalization of the two polymers can still happen in this regime, provided that concentration/affinity of type-1 molecules overcomes a different threshold because type-1 molecules are able to bind to the two polymers and to form bridges between them [9, 10].
Again, the driving physical mechanism here is a thermodynamic phase transition that occurs when the entropy loss due to colocalization is balanced by the energy gain coming from the molecular bridges.
Fig. S5 illustrates the phase diagram of the system as function of the concentrations of the two species of molecules, c_1 and c_2, while all the energies are fixed at the same values ($E_p = E_{12} = E_{22} = 3kT$). It is seen that, if both the types of molecules are present at low concentrations, neither the colocalization nor the symmetry breaking is observed and the polymers independently diffuse in the lattice (gray area, "Independent Diffusion"). When the concentration of type-1 molecules rises above the black dashed line, the polymers do colocalize at equilibrium (green area, "Colocalization"). Yet, to break the symmetry,

it is necessary that type-2 molecules concentration increases until it gets above the red line (orange area, "Symmetry Breaking and Colocalization"). The same scenario above described is observed in a wide range of energies, typical of biochemical interactions (i.e., $E \sim 0 \div 20kT$ [13–18]).

Since such a mechanism relies on robust, general thermodynamic mechanisms, it is not affected by the simplifying assumptions we made to simulate such a complex, many-body system. For instance, the use of directed polymers to represent DNA segments allows faster simulations without affecting the general properties of the colocalization mechanism we describe because they are produced by a general free-energy minimization mechanism, which does not depend on such details. In the case of a non-directed polymer model, DNA sequences would bind to each other as well, but without a perfect alignment as in our model [19].

The results about polymer colocalization discussed in the present model apply to the model we considered in the main text where, analogously, once the molecular aggregates have bound to one of the two polymers, the homologous unbound region on the other can diffuse and become finally bound too. We stress, though, that the occurrence of colocalization, or lack thereof, depends on the specific details of the system. For instance, in the model we discussed in the main text, the case where type-A and B molecules can bind to a polymer bead only if they are not previously bound to other molecules, results in lack of polymer colocalization (although, the discussed conformational modifications and symmetry-breaking effects remain unaltered). Nevertheless, the symmetry breaking mechanism we illustrated can indeed determine the polymer colocalization; yet, as we saw, this occurs on comparatively longer time scales than the symmetry breaking itself, as the colocalization requires the diffusion of whole chromosomes while the symmetry breaking just involves relocation of limited regions within the Xic.

D. Colocalization of X chromosomes at XCI

It has been shown that the two Xic's colocalize approximately when $Xist$ expression is upregulated on one X and downregulated on the other [6–8] . The temporal relation

between Xic colocalization and the structuring of differential intra-chromosomal interactions within the two Xic's is still unexplored. We cannot directly link X colocalization at XCI to the processes described here because in real nuclei a variety of intervening events and complications can arise. However, similar thermodynamic processes [9, 10] could describe the underlying physical mechanisms. And predictions as those emerging from our analysis (e.g., the threshold effect in the concentration/energy of involved molecular factors or in genomic deletions) could be tested against experiments to shed light on the underlying physical processes.

III. EFFECTS OF Xic DELETION/INSERTION ON XCI

In this section we compare the predictions of the model we discussed in the main text with the results of deletion/insertion experiments on XCI reported in the literature. And we show that the model can help rationalizing in a single framework a variety of key experimental data. In our model, for instance, a deletion/insertion of a DNA segment affects X Chromosome Inactivation (XCI) because it modifies the ability of the "Blocking Factor" (BF) or the "Activating Factor" (AF; see main text) to bind the X's. This can happen because the deletion/insertion reduces/increases either the chemical affinity of the molecular components of the AF/BF (by modifying the number of binding sites), or their concentration (if their coding genes are involved in the mutation). To help the non-technical reader, in Fig. S6 we summarize some of the deletions reported in the literature. Below we discuss them along with several other deletions and insertions.

The heterozygous deletion $\Delta 65kb$ [20], also discussed in the Main Text, results to be lethal in males, as it leads to the inactivation of the only X, while in females it always determines the inactivation of the deleted X. The smaller deletions (ΔAS, ΔAJ, ΔAV, $\Delta 34$ [21], $Tsix^{\Delta CpG}$ [22] and $Xite^{\Delta L}$ [23]), nested in the $\Delta 65kb$ deletion, causes the X inactivation in male cells only in a certain fraction of cases. The deletions $Tsix^{\Delta CpG}$ and $Xite^{\Delta L}$ result in the inactivation of the deleted X in heterozygous females. A random choice but a "chaotic" counting is found in homozygous deletions in females, with a number of cells showing two active X chromosomes instead of one. The insertions of these regions in non-sex chromosomes of male cells cause the inactivation of the only X [24–26], while in females can hinder the initiation of XCI [26]. Non-linear effects of insertions have been shown, as long Xic transgenes can cause inactivation on male ES cells only when they are present in multiple copies, while single copies do not have appreciable effects [24].

All these results can be explained by our model, since the deleted areas involve the DNA sites where the binding sites for the BF (which prevents the inactivation of the X it binds to) are mapped (see main text). The longer deletion $\Delta 65kb$ removes a very large portion of the binding sites, resulting in a very low or a zero affinity for the BF with respect to the

Wild Type (WT) X, so it leads to the inactivation of the X that bears the deletion. Yet, the smaller nested deletions are expected to remove a minor fraction of the BF binding sites; so, a "skewed" inactivation is observed in heterozygous deletions, as the BF will preferentially bind to the Wild Type X, protecting it from inactivation. In homozygous deletions the choice is still random because the BF has the same (lower) affinity for the two X's, but it fails to bind in a fraction of cases, determining the "chaotic" counting. The same holds in males: the only X has no competitors for the BF binding, but there is a probability that BF misses its target and that the only X is inactivated. Such a probability will be higher the more the BF binding sites that are removed, so it is expected to increase with the length of the deletion (as it is experimentally observed). The autosomal insertions of these regions in males allow the BF to bind to the autosome, which competes with the X for it, and may leave the real X chromosome prone to inactivation. However, the underlying "switch-like" mechanism of our model, predicts that the probability for a transgene to win the competition for the BF binding can be significant, only if it contains a sufficiently high number of BF binding sites; experiments support this view, by showing the different effects of single/multiple copies insertions of some Xic regions [24]. On the other hand, these regions possibly encode some of the components of the BF itself; thus, their insertion in females, may lead to an overproduction of BF components that could prevent XCI initiation.

The effect of the decrease of *Jpx* RNA molecule concentration is observed to block XCI in female cells, while it has no effect in males [27]. This can be justified within our model, by assuming that *Jpx* RNA molecule enters in the formation of the AF: in this case, the elimination of this molecule may prevent the AF assembling, and consequently, the inactivation of one X.

In turn, the heterozygous deletion of *Jpx*, ΔJpx, has no effect in males, whereas it is found to be lethal for female cells at least in 85% of cases, as the inactivation gets blocked. And, the few female cells which survive this mutation, are shown to preferentially inactivate the Wild Type X chromosome [27]. We saw above that at least some of the binding sites for the AF are mappable around the *Jpx* gene. Thus, an heterozygous deletion of *Jpx* in a female cell could determine an inability of the AF to bind to the mutated X chromosome and to

trigger the inactivation of it. For this reason, a failure of the X inactivation in about the $\sim 50\%$ of cases, when the BF binds to the Wild Type X and the mutated X is designated to get inactivated, is expected. The percentage of failed XCI can be even larger if it is considered that the *Jpx* RNA molecule enters in the formation of the AF, as the above discussed experiment suggests. Summarizing, the ΔJpx deletion, has a double effect which tends to block the inactivation of the X: it hinders the AF assembling because it decreases the concentration of one of its component, and makes the AF binding to the mutated X more difficult (and this explains why the survived cells inactivate more often the Wild Type X).

The absence of major effects of the same deletion in male cells is easily rationalized: as in males the BF is the only molecular complex which binds to the only X (see main text and above), a mutation like ΔJpx which affects the formation/binding of the AF is going to be harmless.

Another very interesting experimental result was obtained by a double heterozygous mutations in females: if the lethal ΔJpx mutation is accompanied by a truncation of $Tsix$ gene on the same X, the cells are able to carry out the X inactivation and survive [27].

In the view of our model, $Tsix$ hosts some binding sites for the BF and is a possible component of the BF itself as a RNA molecule (see above). Thus, the truncation of $Tsix$ gene is expected to affect the BF assembling/binding approximately as the *Jpx* deletion does with the AF. Yet, since according to our symmetry breaking model, the AF or the BF assembling on a X chromosome results from the competition between the respective molecular components on their binding sites, the effect of the double mutation may be different from the mere "sum" of the single mutations effects. In fact, let's consider, for instance the AF: while the ΔJpx reduces the affinity of the AF for the deleted X, the $Tsix$ truncation tends to favour its binding since it reduces the competition due to the BF binding. So, one effect can counterbalance the other and rescue the lethal phenotype of the ΔJpx single mutation.

Additional copies of $Rnf12$ gene have been shown to induce XCI initiation in XY males and on both X's in a substantial percentage of female cells [28]. Conversely, in heterozygous female $Rnf12^{+/-}$ cells, XCI initiation is considerably reduced [28].

Such an experimental picture indicates that $Rnf12$ could encode for an AF component: its high ectopic expression would lead to a higher probability for the AF to bind to the only X in male cells and would hinder the BF formation in female XX cells, by causing respectively, the inactivation of the only X, and the XCI initiation on both X's. On the other hand, the reduced amount of $Rnf12$ transcript in female $Rnf12^{+/-}$, makes the AF assembly more difficult, hence the observed reduction in XCI initiation.

An heterozygous deletion including the promoter of $Xist$ gene, $Xist\Delta promoter$ [29], results in females in the inactivation of the WT X chromosome. The same effect has been observed for the heterozygous deletion $Xist\Delta 1-5$ which involves the first 5 exons of $Xist$ [30], and for the longer deletion ΔXTX (including $Xist$, $Tsix$ and $Xite$ genes [31]).

In the perspective of our model, the two deletions $Xist\Delta promoter$ and $Xist\Delta 1-5$ are likely to cut a number of binding sites for the AF. The AF consequently has a higher affinity for the WT X chromosome in heterozygous XX females: even a small affinity difference in units of kT can produce a substantial difference in the probability of the AF to bind the WT chromosome. Hence, $Xist\Delta promoter$ and $Xist\Delta 1-5$ determine the inactivation of the WT X with very high probability.

The longer ΔXTX deletion removes binding sites for both the AF and for the BF on the deleted chromosome. However, since ΔXTX entails the $Tsix/Xite$ genes, it is likely to reduce the concentration of the BF molecular components. The AF molecules have, thus, a higher probability to win the competition with the BF to bind the wild type X which is then inactivated.

Interestingly, $Xist\Delta 1-5$ and ΔXTX have been also reported to have no effect in males [30, 31]. This is because they remove, at least in part, the $Xist$ gene, and so the only X will remain active.

Transgenic insertions of regions including $Xist$, $Tsix$ and $Xite$ genes (named $\pi JL1$, $\pi JL2$ and $\pi JL3$) have been performed in males as well as in females [26]. While in males such

insertions can determine the initiation of XCI, in females can hinder the inactivation of the X at least in a fraction of cases, as the $Xist$ RNA starts accumulating around the transgene [26].

In our model, if regions spanning the $Tsix$, $Xite$ and $Xist$ genes are introduced in a cell, since they are likely to include binding sites for the AF as well as for the BF, they can interfere in the normal XCI in males, by creating a "false target" for the BF, which can bind to these transgenes leaving the X unprotected from the inactivation. In females, the AF can be bound to this transgene, and determine the observed $Xist$ over-expression on it.

The transgenic insertion of a region located a few kbp upstream of $Xist$ gene, called Xpr [8], has been shown to cause the inactivation of the only X in males and a biallelic expression of $Xist$ gene in females [8]. Both these results can be justified if it is assumed that Xpr codes for a molecule that enters into AF assembling (as also hypothesized in [31]): in fact, the insertion of this region would determine a higher concentration of the AF components, that, in turn, can cause the assembling of an AF on the only X of a male cell and can also delay the BF assembling on one of the two X's in a female cell.

To summarise, in this section we showed how it is possible to rationalize the results of currently available insertion/deletion experiments within the quantitative framework provided by our model. During X Inactivation in real cells a number of additional complications that we do not considered here can intervene. The discussion above supports, though, a picture where the driving mechanism leading to random X inactivation could be controlled by a few core elements acting via simple, general physical processes.

IV. CELLS WITH MORE THAN TWO X'S

It is interesting to speculate about XCI in cells with more than two X's [32]. Our scenario can be extended to describe XCI in those cases, as we describe below, but additional hypotheses must be considered that go beyond our simple physics model because key pieces of biological information are still missing.

For instance in XXXX cells, it is not known whether the X's form, e.g., two independent couples or a single interacting group. This could affect the predictions of our model: in the former case, the most likely event is that an AF and a BF self-assemble in each couple of X's, and so, in most cases, two active and two inactive X would be present in the cell, in agreement with some experimental finding in tetraploid XXXX cells [31]. In the latter case, when the 4X are all in a single interacting group, the symmetry breaking mechanism predicts the formation of two molecular complexes: two X's are bound either to the AF or the BF, whereas the remaining two are depleted of molecular factors and show an open configuration. Thus, the resulting number of active/inactive X's depends on further biological assumptions, e.g., on the state of unbound X's at XCI. And we have to consider that a real population can be a mixture of the above cases. Another complication arising in diploid cells with multiple X's is that the concentration of autosomal products per X chromosome is presumably lower, and this is going to affect the molecular components of the AF and/or BF.

As we have discussed (see above and main text), our model can easily accommodate further layers of complications. Here we prefer, though, to restrict the number of additional hypotheses to a minimum to better serve the purpose to illustrate the basic mechanisms underlying chromatin spatial organization, the focus of the present paper. Conversely, as mentioned before, since the mechanisms for conformational changes we discuss are very robust to difference in molecular details, they could apply to alternative models of XCI in diploid cells with multiple X and polyploid cells.

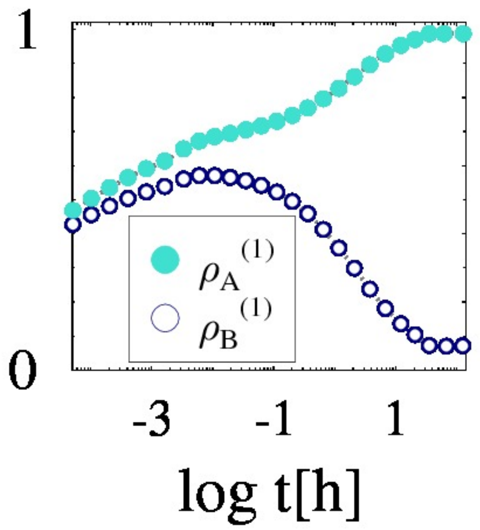

FIG. S1: The density of type-A and B molecules, $\rho_A^{(1)}(t)$ and $\rho_B^{(1)}(t)$, around type-γ region of polymers 1, and the interaction probabilities $p_A^{(1)}(t)$ and $p_B^{(1)}(t)$ of type-α and type-β regions on polymer 1, are shown during a single run of the system dynamics, from an initial symmetrical polymer looped state (see figures in Main Text), for a value of molecule homotypic interaction energy, E_0, in the 'symmetry breaking' phase (here $E_0 = 2kT$, $c = 2\%$ and $E_X = 3kT$). Here, $\rho_A^{(1)}(t)$ grows to one whereas $\rho_B^{(1)}(t)$ goes to zero, and at the same time $p_A^{(1)}(t) \sim 1$ whereas $p_B^{(1)}(t) \to 0$. Thus, while initially molecules are equally distributed around the polymers where both type of loops are formed, after a transient of about ten hours molecules have aggregated around only one of the polymers, and their binding symmetry is broken.

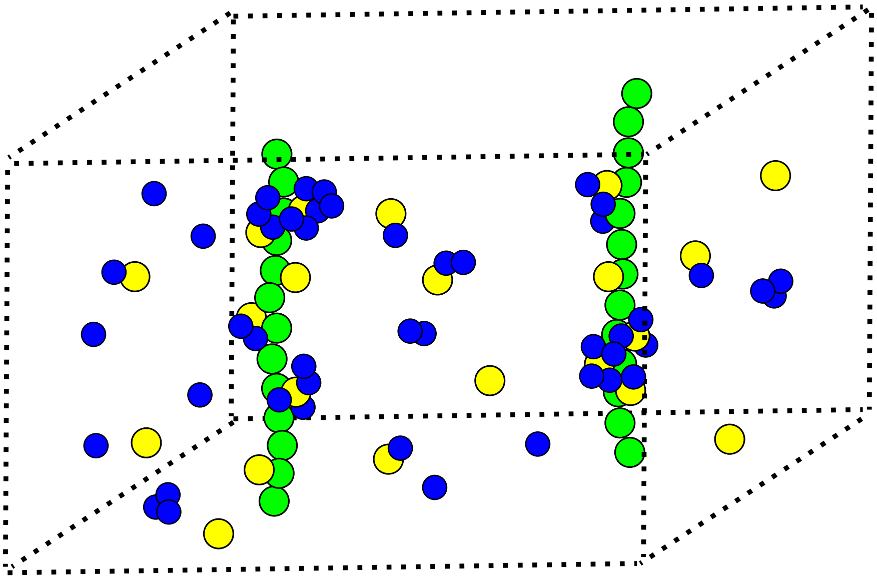

FIG. S2: Pictorial representation of the model system we used to investigate the relationships between the symmetry breaking mechanism and colocalization. In a lattice with periodic boundary conditions, two directed polymers are included with binding sites (green beads) having an affinity E_p for a set of diffusing molecules (type-1 molecules, yellow spheres). These molecules can form bonds of energy E_{12} with another type of molecules (type-2, blue spheres), which, in turn have a reciprocal interaction energy E_{22}.

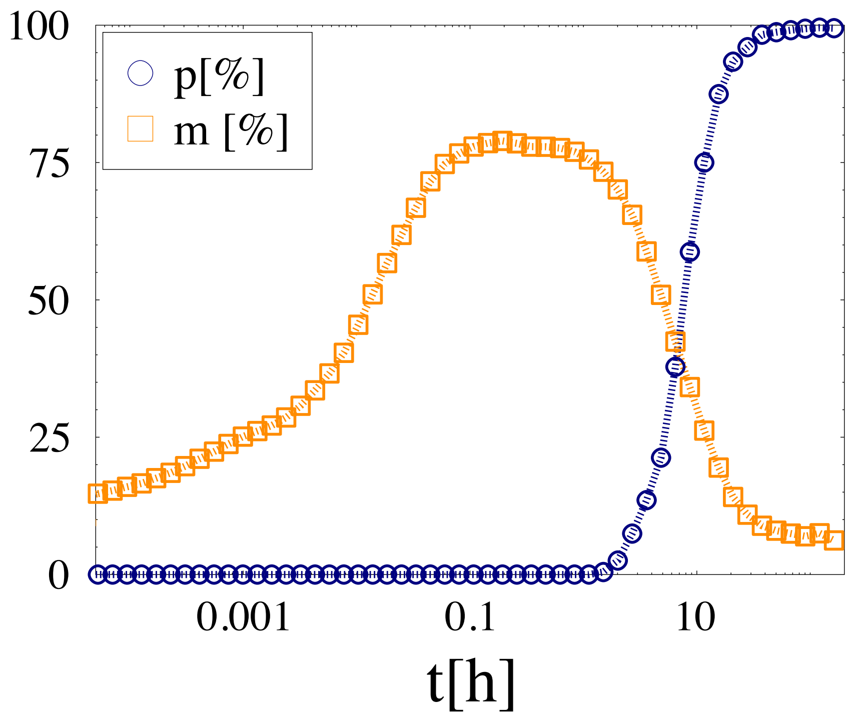

FIG. S3: The pairing probability p of the two polymers (blue circles) and the symmetry-breaking order parameter $m = \left(|\rho_2^{(1)} - \rho_2^{(2)}|\right) / \left(\rho_2^{(1)} + \rho_2^{(2)}\right)$ (orange squares; $\rho_2^{(i)}$ is the density of type-2 molecules around the i-th chromosome) are plotted as function of time. It is shown that the molecules break the binding symmetry between the polymers ($p \sim 0\%$ whereas $m \sim 80\%$ at $t \sim 0.1 \div 1h$); then, in a time scale which is one order of magnitude larger, the polymers do colocalize and single self-assembled molecular cluster includes both of them ($p \sim 100\%$ and $m \sim 0\%$ at $t \gtrsim 10h$). See the snapshots in Fig.S4. Here $E_p = E_{12} = E_{22} = 3kT$, $c_1 = 1\%$ and $c_2 = 4\%$.

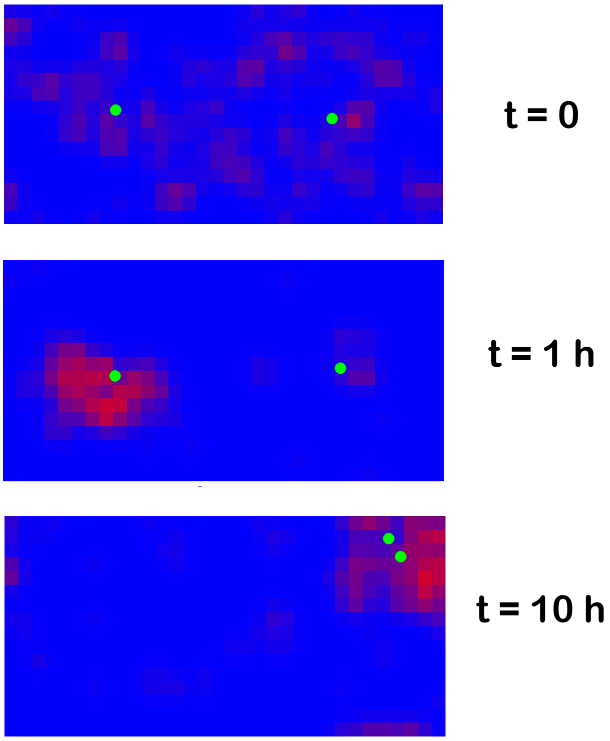

FIG. S4: These 2D projections of the system show the configurations at three different times in a single run, with the polymer center-of-mass marked by green circles and a color map representing the density of type-2 molecules (red color indicates high density regions, blue low density; the values of the parameters are $E_p = E_{12} = E_{22} = 3kT$, $c_1 = 1\%$ and $c_2 = 4\%$). At $t = 0$ (upper panel), the polymers are far apart and the molecules are uniformly distributed in the lattice; at $t = 1h$, the polymers are still far from each other, but a unique molecular cluster is formed around one of the two polymers; finally, at $t = 10h$, the polymers are paired and the molecular cluster includes both of them.

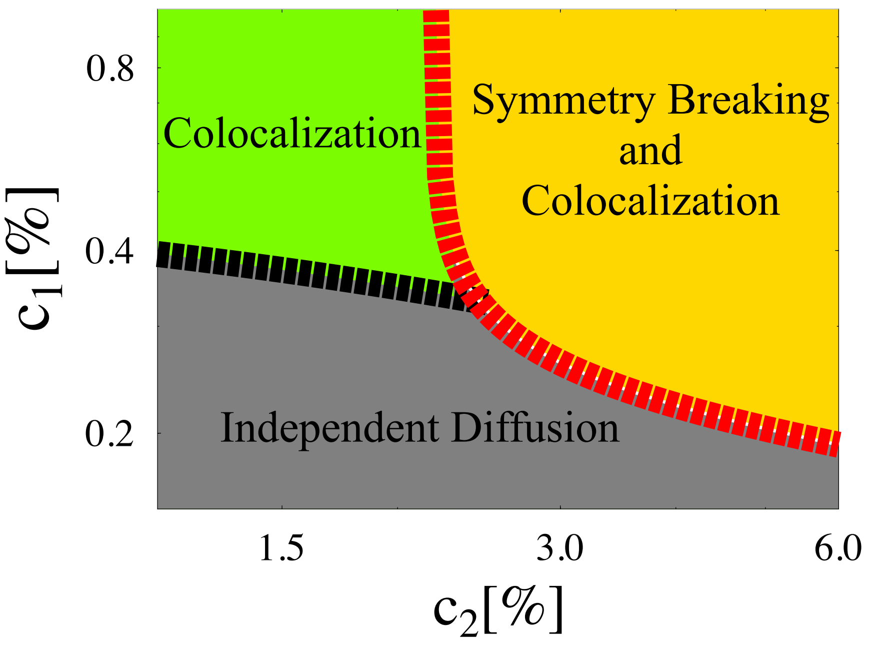

FIG. S5: Phase diagram of the system as function of the concentrations of the two species of molecules, c_1 and c_2, with fixed values of energies ($E_p = E_{12} = E_{22} = 3kT$). At low concentrations, the polymers independently diffuse in the lattice (gray area, "Independent Diffusion"); if c_1 increases above the black dashed line, the polymers stably colocalize at equilibrium ("Colocalization"); the symmetry breaking occurs when c_2 increases and the red dashed line is passed.

Name	Genotype
Wild Type	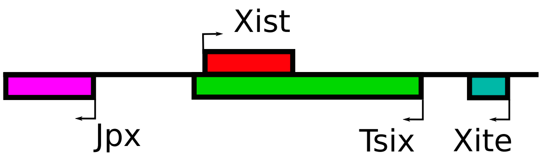
$\Delta 65kb$	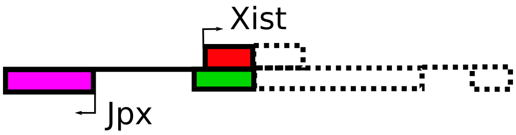
$Tsix^{\Delta CpG}$	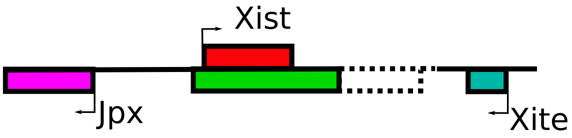
ΔXTX	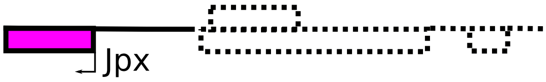
ΔJpx	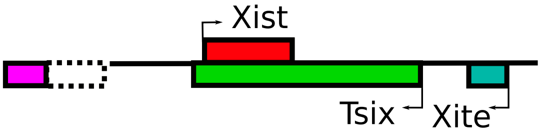

FIG. S6: The table summarizes the genotype of some of the deletions we discuss in the text to help visualizing their features and effects. From top to bottom, we show a drawing of the Wild-Type Xic locus and then: $\Delta 65kb$ [20], $Tsix^{\Delta CpG}$ [22], ΔXTX [31] and ΔJpx [27]

[1] Doi M, Edwards S (1986) *The Theory of Polymer Dynamics* (Clarendon Press, Oxford, USA).

[2] Donohoe ME, Zhang LF, Xu N, Shi Y, Lee JT (2007) Identification of a Ctcf cofactor, Yy1, for the X chromosome binary switch. *Mol Cell* 25:43–56.

[3] Binder K (1997) Applications of Monte Carlo methods to statistical physics. *Reports on Progress In Physics* 60:487–559.

[4] Chubb JR, Boyle S, Perry P, Bickmore WA (2002) Chromatin motion is constrained by association with nuclear compartments in human cells. *Current Biology* 12:439–45.

[5] Watson JD, et al. (2003) *Molecular Biology of the Gene* (Benjamin Cummings), 5th edition.

[6] Bacher CP, et al. (2006) Transient colocalization of X-inactivation centres accompanies the initiation of X inactivation. *Nat Cell Biol* 8:293–299.

[7] Xu N, Tsai CL, Lee JT (2006) Transient homologous chromosome pairing marks the onset of X inactivation. *Science* 311:1149–1152.

[8] Augui S, et al. (2007) Sensing X chromosome pairs before X Inactivation via a novel X-pairing region of the Xic. *Science* 318:1632–1636.

[9] Nicodemi M, Panning B, Prisco A (2008) A thermodynamic switch for chromosome colocalization. *Genetics* 179:717–21.

[10] Scialdone A, Nicodemi M (2008) Mechanics and dynamics of X-chromosome pairing at X Inactivation. *PLoS Comput Biol* 4:e1000244.

[11] Phair RD, Misteli T (2000) High mobility of proteins in the mammalian cell nucleus. *Nature* 404:604–9.

[12] Chandler D (1987) *Introduction to Modern Statistical Mechanics* (Oxford University Press).

[13] Berg J (2008) Dynamics of gene expression and the regulatory inference problem. *Europhys. Lett.* 82:28010.

[14] Gerland U, Moroz JD, Hwa T (2002) Physical constraints and functional characteristics of transcription factor-dna interaction. *Proc Natl Acad Sci U S A* 99:12015–12020.

[15] Lassig M (2007) From biophysics to evolutionary genetics: statistical aspects of gene regulation. *BMC Bioinformatics* 8 Suppl 6:S7.

[16] Maerkl SJ, Quake SR (2007) A systems approach to measuring the binding energy landscapes of transcription factors. *Science* 315:233–7.

[17] Massie C, Mills I (2008) Chipping away at gene regulation. *EMBO Rep. Review* 9:337–43.

[18] Morozov AV, Havranek JJ, Baker D, Siggia ED (2005) Protein-DNA binding specificity predictions with structural models. *Nucleic Acids Res* 33:5781–98.

[19] Nicodemi M, Prisco A (2009) Thermodynamic pathways to genome spatial organization in the cell nucleus. *Biophys J* 96:2168–2177.

[20] Clerc P, Avner P (1998) Role of the region 3' to Xist exon 6 in the counting process of X-chromosome inactivation. *Nat Genet* 19:249–53.

[21] Vigneau S, Augui S, Navarro P, Avner P, Clerc P (2006) An essential role for the DXPas34 tandem repeat and Tsix transcription in the counting process of X chromosome inactivation. *Proc Natl Acad Sci U S A* 103:7390–5.

[22] Lee JT, Lu N (1999) Targeted mutagenesis of Tsix leads to nonrandom X inactivation. *Cell* 99:47–57.

[23] Ogawa Y, Lee JT (2003) Xite, X-Inactivation intergenic transcription elements that regulate the probability of choice. *Mol Cell* 11:731–43.

[24] Heard E, Mongelard F, Arnaud D, Avner P (1999) Xist yeast artificial chromosome transgenes function as X-inactivation centers only in multicopy arrays and not as single copies. *Mol. Cell. Biol.* 19:3156–3166.

[25] Lee JT, Strauss WM, Dausman JA, Jaenisch R (1996) A 450 kb transgene displays properties of the mammalian X-inactivation center. *Cell* 86:83–94.

[26] Lee JT (2005) Regulation of X-chromosome counting by Tsix and Xite sequences. *Science* 309:768–71.

[27] Tian D, Sun S, Lee JT (2010) The long noncoding RNA, Jpx, is a molecular switch for X chromosome inactivation. *Cell* 143:390–403.

[28] Jonkers I, et al. (2009) RNF12 is an X-Encoded dose-dependent activator of X chromosome inactivation. *Cell* 139:999–1011.

[29] Penny GD, Kay GF, Sheardown SA, Rastan S, Brockdorff N (1996) Requirement for xist in x chromosome inactivation. *Nature* 379:131–7.

[30] Marahrens Y, Panning B, Dausman J, Strauss W, Jaenisch R (1997) Xist-deficient mice are defective in dosage compensation but not spermatogenesis. *Genes Dev* 11:156–66.

[31] Monkhorst K, Jonkers I, Rentmeester E, Grosveld F, Gribnau J (2008) X Inactivation counting and choice is a stochastic process: evidence for involvement of an X-linked activator. *Cell*

132:410–421.

[32] Bianco V, Scialdone A, Nicodemi M (2011) *submitted*.
